# Exacerbation of neuronal senescence after spinal cord injury: Role of the macrophage-derived transforming growth factor-β1–SMAD2 signaling axis

**DOI:** 10.4103/NRR.NRR-D-24-01376

**Published:** 2025-06-19

**Authors:** Haiwen Feng, Hongda Wang, Junjin Li, Jie Ren, Yuanquan Li, Chuanhao Li, Junyu Chen, Xiaomeng Song, Guangzhi Ning, Shiqing Feng

**Affiliations:** 1 Tianjin Key Laboratory of Spine and Spinal Cord, International Science and Technology Cooperation Base of Spinal Cord Injury, Department of Orthopedics, International Chinese Musculoskeletal Research Society Collaborating Center for Spinal Cord Injury, Tianjin Medical University General Hospital, Tianjin, China; 2 Orthopedic Research Center of Shandong University and Department of Orthopedics, Qilu Hospital of Shandong University, Cheeloo College of Medicine, Shandong University, Jinan, Shandong Province, China

**Keywords:** cellular senescence, macrophage, neural regeneration, neurodegenerative disease, neuroinflammation, neuron, neuronal repair, spinal cord contusion, spinal cord injury, TGF-β1–SMAD2

## Abstract

Neuronal degeneration and inflammation are hallmark features of spinal cord injury that severely hinder functional recovery. As key regulators of the post-injury microenvironment, macrophages can promote either tissue repair or exacerbate damage. Among macrophage secreted factors, transforming growth factor-beta 1 has emerged as a critical mediator of pathological changes. In this study, we show the pivotal role of macrophage-derived transforming growth factor-beta 1 in driving neuronal senescence and impairing functional recovery after spinal cord injury. In a mouse spinal cord injury model, transforming growth factor-beta 1 levels were significantly increased at the injury site, accompanied by increased mothers against decapentaplegic homolog 2 (SMAD2) phosphorylation and upregulation of neuronal senescence markers such as p16^INK4a^ and β-galactosidase activity. Treatment with LY-364947, a SMAD2 phosphorylation inhibitor, markedly reduced the number of senescent neurons, mitigated tissue degeneration, and improved motor function recovery. Additionally, macrophage depletion using clodronate liposomes lowered transforming growth factor-beta 1 levels at the injury site and attenuated neuronal senescence. These findings highlight the transforming growth factor-beta 1–SMAD2 signaling axis as a potential therapeutic target to reduce neuronal senescence and enhance functional recovery following spinal cord injury.

## Graphical abstract

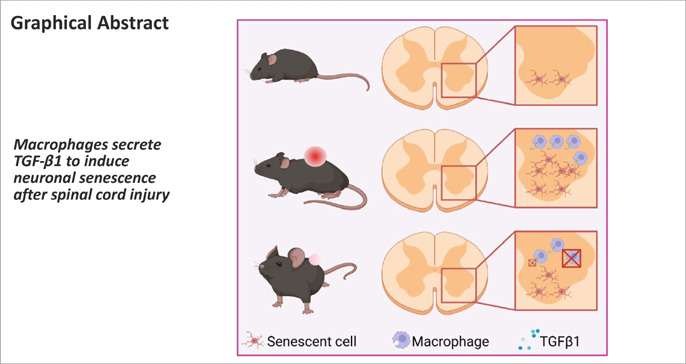

## Introduction

Spinal cord injury (SCI) of the central nervous system significantly impacts the physical and psychological wellbeing of patients (Furlan et al., 2011; Huang et al., 2020). Following SCI, stress-induced alterations in cellular states and functions disrupt the microenvironment. These are considered to be key pathological mechanisms hindering SCI recovery, as cells exposed to stress conditions have the potential to enter a state of senescence (Fan et al., 2018). Cellular senescence has been primarily associated with chronic diseases. However, an increasing body of evidence indicates that cellular senescence plays a critical role in injury processes and may have long-lasting effects (Faust et al., 2020).

Immune cells play a pivotal role in driving the senescence of functional cells in the central nervous system (Feng et al., 2024). For instance, in the brain, natural killer cells promote neural progenitor cell senescence through interleukin (IL)-17 secretion, thereby impairing cognitive function (Sarig et al., 2019). Similarly, in primates, chitotriosidase 1-positive (CHIT1^+^) microglia can induce motor neuron senescence via CHIT1 secretion, leading to motor dysfunction (Sun et al., 2023). The roles of cellular senescence and neuroimmunity are crucial to central nervous system function. Although previous studies have extensively explored the mechanisms of neuroimmune responses in SCI (Sterner and Sterner, 2022), particularly the roles of macrophages and microglia (Fan et al., 2020), research on the impact of cellular senescence in SCI remains limited.

Cellular senescence is characterized by cell cycle arrest, manifested through cell cycle inhibitor protein P16INK4α upregulation (Beauséjour et al., 2003) and cytoplasmic granule accumulation due to enhanced lysosomal hydrolase activity (Lee et al., 2006). Senescent cell accumulation leads to a decline in normal tissue function (Coppé et al., 2010). Moreover, an increase in senescent cells within the spinal cord can impair its normal function and increase the risk of spinal cord pathology (Roberts, 1990). An increase in senescent motor neurons is observed in the spinal cord of aged primates and mice, with studies indicating that motor neuron senescence is associated with mothers against decapentaplegic homolog 2 (SMAD2) phosphorylation within these cells. Furthermore, in primates, CHIT1^+^ microglia activate SMAD2 phosphorylation through CHIT1 secretion. In mice, CHIT1^+^ microglia have not been observed (Sun et al., 2023). Yet senescent neuron accumulation was found in mice following SCI, and the clearance of senescent neurons with Navitoclax (ABT-263) had neuroprotective effects (Paramos-de-Carvalho et al., 2021). Nevertheless, the mechanisms underlying neuronal senescence remain poorly understood and require further investigation.

Transforming growth factor-β1 (TGF-β1) plays a critical regulatory role in the proliferation, survival, and apoptosis of various cells in the nervous system (Meyers and Kessler, 2017). After SCI, macrophage activity is crucial for neural repair. In the acute and subacute phases (< 7 days), M1 macrophages predominate, while M2 macrophages become the dominant subtype in the chronic phase (> 7 days) (Milich et al., 2019). M2 macrophages secrete significant amounts of TGF-β1 (Kigerl et al., 2009). Following liver injury, hepatocyte senescence is primarily driven by macrophage-derived TGF-β1, which activates SMAD2 phosphorylation pathways within hepatocytes (Bird et al., 2018). This suggests that TGF-β1 produced after SCI may be a potential factor contributing to the senescence of motor neurons.

The role of SMAD signaling pathway in cellular senescence has garnered increasing attention (Zhang et al., 2020). Studies have shown that SMAD pathway activation upregulates cell cycle inhibitory gene expression, such as p16, p21, and p27, leading to cell cycle arrest and inducing cells to enter a senescent state (Yuan et al., 2022; Yu et al., 2024). This correlation between SMAD pathway activation and cellular senescence is observed in models of liver injury (Bird et al., 2018), as well as during embryonic development (Di Micco et al., 2021). As a downstream pathway of TGF-β1, SMAD signaling is activated in cells at the site of SCI because of TGF-β1 accumulation. This mechanism provides new insights into the regulatory role of signaling pathways in cellular senescence and injury repair.

To confirm that macrophage-derived TGF-β1 induces neuronal senescence via SMAD2 protein phosphorylation, we administered LY-364947 (a SMAD2 phosphorylation inhibitor; Zhang et al., 2022) and clodronate liposomes (a macrophage depletion agent; Xiong et al., 2022) to mice from 5 to 28 days following SCI. This administration time was determined based on a previous study showing increased senescent cell accumulation at this time point (Paramos-de-Carvalho et al., 2021). Our study aimed to investigate the causes of senescent cell accumulation following SCI and determine whether intervening to reduce senescent cells contributes to the repair of SCI.

## Methods

### Ethics statement

This study was approved by the Animal Welfare and Ethics Committee of Tianjin Medical University General Hospital (approval No. IRB2024-SW-46) on June 7, 2024. All procedures strictly adhered to the AVMA Guidelines for the Euthanasia of Animals to minimize distress (American Veterinary Medical Foundation [AVMF], 2020). Mice were supplied by Beijing Vital River Laboratory Animal Technology Co., Ltd. (animal license No. SCXK (Jing) 2021-0006). Surgical anesthesia was induced and maintained via isoflurane (RWD, Shenzhen, China) inhalation (5% for induction and 2% for maintenance). At the experimental endpoint, we euthanized the mice by CO_2_ asphyxiation at a displacement rate of 30% chamber volume per minute, followed by cervical dislocation to confirm death.

### Cell culture and intervention

HT22 cells (mouse hippocampal neuronal cells) were purchased from Procell Life Science & Technology Co., Ltd. (Wuhan, China; Cat# CL-0697) and authenticated by short tandem repeat analysis. The cells were cultured at 37°C in a humidified atmosphere with 5% CO_2_ in 90% high-glucose Dulbecco’s modified Eagle medium (Gibco, New York, NY, USA) supplemented with 10% fetal bovine serum (Gibco). HT22 cells were adjusted to a concentration of 5 × 10^4^/mL and seeded in 6- or 24-well plates for 24 hours. Cells were incubated with 50 µM hydrogen peroxide (H_2_O_2_) to establish an oxidative stress model. Cells were treated with LY-364947 (3 µM, MedChemExpress, Newark, NJ, USA) and/or TGF-β1 (5 ng/mL, Novoprotein, Suzhou, China). Cells in 24-well plates were used for senescence-associated β-galactosidase (SA-β-gal) staining or immunofluorescence analysis, while cells in 6-well plates were used for protein extraction and subsequent western blot analysis.

Mouse macrophages were isolated from the bone marrow of female C57BL/6J mice (aged 6–8 weeks; weight 18–20 g). Macrophages were cultured at 37°C in a humidified atmosphere with 5% CO_2_ in 90% high-glucose Dulbecco’s modified Eagle medium (Gibco) supplemented with 10% fetal bovine serum and 20 ng/mL M-CSF (Sigma, Darmstadt, Germany).

Macrophages were identified by flow cytometry (**Additional Figure 1A**), adjusted to a concentration of 5 × 10^4^/mL and seeded in 6-well plates for 24 hours. IL-4 is a key cytokine that promotes the differentiation of macrophages into the M2 phenotype (Lawrence and Natoli, 2011), therefore, it was used to mediate macrophage M2 polarization. The cells were treated under different interventions, as follows: control group (no treatment) and IL-4 (10 ng/mL, GenScript, Piscataway, NJ, USA) treatment group (**Additional Figure 2**).

### Mice and surgical procedure

To minimize intra- and post-operative stress responses in experimental animals and optimize SCI research, female C57BL/6J mice were selected. Mice were acclimated to the housing environment for 1 week prior to surgery (Skinnider et al., 2024). The anesthesia used was a mixture of isoflurane (2%) and oxygen delivered through a mask. The experimental animals were randomly divided into four groups: sham group, SCI group, SCI + LY-364947 group, and SCI + clodronate liposome group, with 10 animals in each group. For the experimental procedure, the ninth thoracic vertebra (T9) was removed (Ran et al., 2023). In the sham group, only the muscle and skin were sutured without SCI. In the injury group, the mice received a spinal cord impact using the Center Impact Models AIII (W.M. Keck, Hawaii, USA). A specially designed impactor tip for mice (weighing 10 g, diameter of 1 mm) was used. After adequately exposing the spinal cord, the impactor was aligned with the center of the spinal cord, and the impactor head positioned 12.5 mm above the cord. The impact was then delivered. Post-impact, hind limb twitching and hematoma formation at the injury site were observed. During the experimental period, the researchers manually expressed the bladder twice daily.

### Drug administration

From days 5 to 28 post-surgery, mice in the sham and SCI groups received daily intraperitoneal injections of 100 µL of normal saline. Mice in the LY-364947 group were administered 100 µL of LY-364947 (1 mg/kg; dissolved in normal saline) by daily intraperitoneal injections, while those in the clodronate liposome group received intraperitoneal injections of 100 µL of clodronate liposomes (YEASEN, Shanghai, China; 40337) every 3 days (**Additional Figure 2**). Clodronate liposomes are used to deplete macrophages because they contain clodronate, a bisphosphonate that induces cell death when delivered inside macrophages (Song et al., 2019).

### Western blotting

Mice were anesthetized using 2% isoflurane via inhalation, and after confirmation of deep anesthesia, were sacrificed by cervical dislocation, followed by spinal cord tissue collection (2 mm from the injury site). HT22 cells or spinal cord tissue samples (2 mm in length) were lysed in lysis buffer containing protease and phosphatase inhibitors (Beyotime, P0013, Shanghai, China). After homogenization, the lysates were centrifuged at 12,000 × *g* for 15 minutes and supernatants were collected. Supernatants were diluted with sodium dodecyl sulfate–polyacrylamide gel electrophoresis loading buffer (Epizyme Biotech, Cambridge, MA, USA, AT101) and heated at 100°C for 5 minutes to denature proteins. Protein separation was performed using either 10% or 12.5% sodium dodecyl sulfate–polyacrylamide gel electrophoresis gels. After electrophoresis, proteins were transferred onto 0.2 µm polyvinylidene fluoride membrane, which was blocked with 5% non-fat milk at room temperature for 1 hour. Subsequently, membranes were incubated overnight at 4°C with primary antibodies, including anti-SMAD2/3 (rabbit, 1:1000, Cell Signaling, Boston, MA, USA, Cat# 8685, RRID: AB_10889933), anti-phosphorylated SMAD2 (rabbit, 1:1000, Cell Signaling, Cat# 18338, RRID: AB_2798798), anti-TGF-β1 (rabbit, 1:1000, Abcam, Cambridge, UK, Cat# ab215715, RRID:AB_2893156), and anti-glyceraldehyde-3-phosphate dehydrogenase (rabbit, 1:1000, Proteintech, Wuhan, China, Cat# 10494-1-AP, RRID: AB_2263076). The membrane was then incubated with horseradish peroxidase-conjugated secondary antibody (goat, 1:1000, Beyotime, Cat# A0208, RRID: AB_2892644) at room temperature for 1 hour. To visualize protein bands, the membrane was treated with chemiluminescence substrate (Millipore, Billerica, MA, USA, EBKLS) and imaged using a chemiluminescence detection system (CLINX 6300, Qinxiang, Shanghai, China). Quantitative analysis of immunoblot images was performed using ImageJ 1.54g software (National Institutes of Health, Bethesda, MD, USA) (Schneider et al., 2012). Relative expression levels of each target protein were obtained by normalizing to glyceraldehyde-3-phosphate dehydrogenase.

### Immunofluorescence staining

Spinal cord coronal sections (10 μm) were prepared using a cryostat (Leica, Wetzlar, Germany, CM3050S). For gastrocnemius muscle, paraffin sections (10 μm) were prepared along the midline using a paraffin microtome. HT22 cells cultured in 24-well plates were fixed with 4% paraformaldehyde for 30 minutes after removing culture medium. Spinal cord sections (either directly or after β-galactosidase staining) and HT22 cells were blocked with immunostaining blocking solution for 1 hour. This was followed by overnight incubation at 4°C with primary antibodies, including anti-P16 (rabbit, 1:500, Proteintech, Cat# 10883-1-AP, RRID: AB_2078303), anti-phosphorylated SMAD2 (rabbit, 1:500, Cell Signaling, Cat# 8685, RRID: AB_10889933), anti-NeuN (mouse, 1:500, Abcam, Cat# ab177487, RRID: AB_2532109), anti-glial fibrillary acidic protein (GFAP; rabbit, 1:500, Abcam, Cat# ab7260, RRID: AB_305808), anti-neurofilament 200 (NF200; mouse, 1:500, Sigma, Cat# N5389, RRID: AB_260781), anti-myelin basic protein (MBP; rabbit, 1:500, Abcam, Cat# ab76109, RRID: AB_1310460), and anti-lamin (rabbit, 1:500, Abcam, Cat# ab133645, RRID: AB_2877125). Sections or cells were then incubated with secondary antibodies (Alexa Fluor 488, goat, 1:500, Proteintech, Cat# SA00013-1, RRID: AB_2810983; Alexa Fluor 488, goat, 1:500, Proteintech, Cat# SA00013-2, RRID: AB_2797132; Cy3, goat, 1:500, Proteintech, Cat# SA00009-1, RRID: AB_2814746; and Cy3, goat, 1:500, Proteintech, Cat# SA00009-2, RRID: AB_2890957) at room temperature for 1 hour. Finally, 4,6-diamidino-2-phenylindole (Abcam, ab5390) was applied for nuclear staining, and the samples mounted. Fluorescence images were acquired using a fluorescence microscope (Leica, Dmi8), and quantitative image analysis was performed using ImageJ software.

### β-Galactosidase staining

Spinal cord sections (10 μm) were prepared using a cryostat. For HT22 cells cultured in 24-well plates, the culture medium was removed and then cells were fixed with 4% paraformaldehyde for 30 minutes. β-Galactosidase staining was performed using a β-galactosidase staining kit (Solarbio, Beijing, China, G1580) (Xiong et al., 2024). β-Galactosidase fixative was applied to tissue sections or cells and fixed at room temperature for 15 minutes. Sections were washed three times with phosphate-buffered saline (PBS) (Gibco) for 5 minutes each. Staining solution was then added. Sections were incubated overnight at 37°C, while cells were incubated at 37°C for 48 hours. To prevent evaporation of the staining solution, culture plates were sealed with plastic wrap. After staining, tissue sections were prepared for the next step, while cells were observed under a standard light microscope (Olympus, Tokyo, Japan, IX73).

### Hematoxylin and eosin staining

After SA-β-gal staining, spinal cord and bladder tissue sections were stained using a hematoxylin and eosin (H&E) staining kit (Solarbio, G1120). First, tissue sections were brought to room temperature from –20°C, followed by washing with 0.3 M glycine for 5 minutes to neutralize residual paraformaldehyde. Sections were then washed three times using tris-buffered saline with Tween 20 for 5 minutes each, fixed with 4% paraformaldehyde for 30 seconds, and rinsed with distilled water for 1 minute. Next, sections were immersed in hematoxylin staining solution for 3 minutes and then rinsed with water to remove excess stain. Slides were briefly dipped in 1% hydrochloric acid–ethanol for 2 seconds, followed by a 1–2-second rinse with water. Nuclei appeared bluish-purple under the microscope. Sections were then immersed in bluing reagent for 5–10 seconds, briefly rinsed in water for 1–2 seconds, stained with eosin solution for 30–60 seconds, and rinsed with water. Finally, sections were mounted with neutral resin and observed under a standard light microscope.

### Gait analysis

The CatWalk 9.0 gait analysis system (Noldus, Wageningen, Netherlands) was used to examine hind limb motor function and coordination in mice (Ren et al., 2023). Prior to the experiment, the camera was positioned 11 cm above the runway to ensure clear capture of paw prints. For 3 days before formal gait analysis, each mouse underwent 30-minute daily training sessions on the CatWalk runway. The runway width was adjusted to allow only passage of the mouse, preventing them from turning around. Mice were trained to run at a consistent speed towards a dark box. A successful trial was defined as completing the task without interruption. During the formal experiment, each mouse was assigned an identification number in the system and required to complete three consecutive runs. The positions of the four limbs were marked, and the average of three runs was used for data analysis and evaluation.

### Electrophysiological assessment

Motor evoked potentials were recorded using an electrophysiological detection system (YRKJ-G2008, Yiruikeji Co., Guangzhou, China) to determine recovery of motor function in mice prior to tissue collection (Gu et al., 2023). Before testing, mice were anesthetized with isoflurane. Then the scalp, back, and both thigh areas were shaved and disinfected with 75% alcohol. After assembling and connecting electrophysiological equipment, conductive gel was applied to electrode needles. The stimulation electrode was placed beneath the scalp, the ground electrode on the back, and the reference and recording electrodes were inserted into the gastrocnemius muscles of both thighs. The stimulation intensity and frequency were adjusted according to the experimental requirements. Each mouse underwent three successful waveform acquisitions, and the average value was used to analyze and calculate the motor evoked potential latency and amplitude across different groups.

### Bladder ultrasound

Four weeks after SCI, the residual bladder volume in mice was measured using a small animal ultrasound system (VisualSonics, Vevo2100, Toronto, Canada) (Xiao et al., 2023). The skin over the pubic region was prepared. Mice were anesthetized with isoflurane and positioned in a supine position on the examination platform. A small amount of ultrasound gel was applied to the pubic area, and the ultrasound probe placed on top of the gel. Bladder images were acquired in both sagittal and transverse planes, and the maximum bladder width, length, and depth were measured. The residual urine volume was then calculated using the prolate ellipsoid volume formula.

### Flow cytometry

Cultured macrophages were harvested using trypsin digestion, followed by washing and centrifugation to remove the supernatant. Cells were then resuspended in fluorescence-activated cell sorting (FACS) buffer. Zombie UV^TM^ Fixable Viability Kit (BioLegend, San Diego, CA, USA) was used to stain cells. Next, to determine macrophage M2 polarization, the cells were suspended in FACS buffer for surface antibody staining, including CD45 (BioLegend, 103132), CD11b (BioLegend, 101251), F4/80 (BioLegend, 123107), CD206 (BioLegend, 141733), and Ly6G (BioLegend, 127613). After antibody staining, the samples were fixed with 1% paraformaldehyde and analyzed using a flow cytometer (BD LSRFortessa, Franklin Lakes, NJ, USA).

Approximately 1-mm^3^ tissue blocks were excised from the center and adjacent areas of the SCI site, and dissociated using Neural Tissue Dissociation Kit (Miltenyi Biotec, Bergisch Gladbach, Germany, 130-092-628). Dissociated cells were washed with Debris Removal Solution (Miltenyi Biotec, Bergisch Gladbach, Germany, 130-109-398) and centrifuged at 3000 × *g* for 10 minutes to remove debris. To determine the retention of macrophages at the epicenter and surrounding areas post-SCI, cells were resuspended in FACS buffer and stained with Zombie UV^TM^ Fixable Viability Kit, followed by antibody staining for CD45 (BioLegend, 103132), CD11b (BioLegend, 101251), F4/80 (BioLegend, 123107), and Ly6G (BioLegend, 127613). After antibody staining, the samples were fixed with 1% paraformaldehyde and analyzed using a flow cytometer.

### Enzyme-linked immunosorbent assay

The TGF-β1 concentration in macrophage supernatants was measured using a mouse TGF-β1 enzyme-linked immunosorbent assay kit (Proteintech, KE10005) (Yu et al., 2024). Supernatants from cultured macrophages of different groups were collected and centrifuged at 500 × *g* for 5 minutes to obtain clear supernatants. The enzyme-linked immunosorbent assay was subsequently performed following the manufacturer’s protocol, and the optical density of each well was measured at 450 nm using a microplate reader (BioTek, Winooski, VT, USA). TGF-β1 concentrations were calculated using a four-parameter logistic curve fitting method in Origin 2023b (OriginLab Corporation, Northampton, MA, USA; Priyadarshi et al., 2013), with the sample optical density interpolated from the standard curve.

### RNA sequencing

Total RNA from spinal cord samples of mice in the SCI and LY-364947 groups was extracted and purified using TRIzol reagent (Thermo Fisher Scientific, Waltham, MA, USA, 15596018). RNA samples were sequenced using the Illumina NovaSeq^TM^ 6000 platform (LC-Bio Technology Co., Ltd., Hangzhou, China). Kyoto Encyclopedia of Genes and Genomes (KEGG), Gene Ontology (GO), and Gene Set Enrichment Analysis (GSEA) were performed based on RNA sequencing data using normalized gene expression values after rigorous filtering. KEGG and GO pathway enrichment analyses were conducted using the ClusterProfiler package (www.lc-bio.com), with thresholds set at *P* < 0.05 and *q* < 0.05. To calculate enrichment scores for predefined gene sets, GSEA analysis was performed using a normalized gene expression matrix with relevant software (www.lc-bio.com), with a significance threshold of *P* < 0.05. All analyses were conducted with default parameters and the results were interpreted in conjunction with relevant literature.

### Statistical analysis

The minimum sample size per group was determined by power analysis (α = 0.05, 1 – β = 0.8, two-tailed tests), resulting in a requirement of 10 mice per group. To account for potential attrition, 12 mice were ultimately enrolled in each group. During the experiment, two animals were excluded due to post-operative infections and were replaced following ethical approval. All behavioral assessments were performed by investigators blinded to group allocation. Data recording used a coded blinding system to ensure unbiased analysis.

All data are expressed as mean ± standard error of the mean (SEM). Comparisons between the two groups were made using unpaired two-tailed Student’s *t*-test, while comparisons among three groups were performed using one-way analysis of variance followed by Tukey’s *post hoc* test, with *P*-values < 0.05 considered statistically significant. Statistical analyses included rigorous normality and variance tests. All statistical analyses were performed using GraphPad Prism software (version 10.1.2 for Windows, GraphPad Software, Boston, MA, USA, www.graphpad.com).

## Results

### LY-364947 inhibits H_2_O_2_- and transforming growth factor-beta 1-induced senescence phenotype in HT22 cells

**Figure 1:**
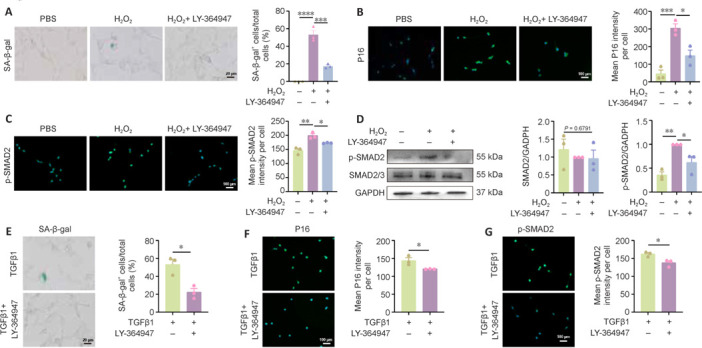
LY-364947 inhibits H_2_O_2_- and TGF-β1-induced senescence phenotype in HT22 cells. (A) Representative images and quantitative analysis of β-galactosidase staining in HT22 cells under different culture conditions (*n* = 3). Scale bar: 20 μm. (B) Representative images and quantitative analysis of P16 immunofluorescence staining in HT22 cells under different culture conditions (*n* = 3). Scale bar: 100 μm. (C) Representative images and quantitative analysis of p-SMAD2 immunofluorescence staining in HT22 cells under different culture conditions (*n* = 3). Scale bar: 100 μm. (D) Representative images and quantitative analysis of western blot analysis for SMAD2/3 and p-SMAD2 in HT22 cells under different culture conditions (*n* = 3). (E) Representative images and quantitative analysis of β-galactosidase staining in HT22 cells under different culture conditions (*n* = 3). Scale bar: 20 μm. (F) Representative images and quantitative analysis of P16 immunofluorescence staining in HT22 cells under different culture conditions (*n* = 3). Scale bar: 100 μm. (G) Representative images and quantitative analysis of p-SMAD2 immunofluorescence staining in HT22 cells under different culture conditions (*n* = 3). Scale bar: 100 μm. Data are expressed as mean ± SEM. *P < 0.05, **P < 0.01, ***P < 0.001, ****P < 0.0001 (two-tailed Student’s *t*-test [E–G] or one-way analysis of variance followed by Tukey’s *post hoc* test [A–D]). H_2_O_2_: Hydrogen peroxide; LY-364947: a SMAD2 phosphorylation inhibitor; PBS: phosphate buffered saline; p-SMAD2: phospho-SMAD2; SA-β-gal: senescence-associated β-galactosidase; SMAD2/3: mothers against decapentaplegic homolog 2/3; TGF-β1: transforming growth factor-β1.

Low concentrations of H_2_O_2_ were applied to HT22 cells *in vitro* as a model to induce neuronal senescence (Ran et al., 2023). Following H_2_O_2_ treatment, the proportion of SA-β-gal-positive HT22 cells increased significantly, indicating successful cellular senescence induction. However, intervention with the SMAD2 phosphorylation inhibitor, LY-364947, reduced the proportion of SA-β-gal-positive cells (**Figure 1A**). Immunofluorescence staining further revealed significant upregulation of P16 protein expression in H_2_O_2_-treated cells, which was attenuated by LY-364947 (**Figure 1B**). These findings suggest that low concentrations of H_2_O_2_ can induce neuronal senescence, while inhibiting SMAD2 phosphorylation mitigates this effect.

To further investigate the role of the TGF-β1–SMAD2/3 pathway in neuronal senescence, immunofluorescence and western blot analyses were conducted to examine SMAD2 phosphorylation levels. The results showed a marked increase in SMAD2 phosphorylation expression in neurons treated with H_2_O_2_ (**Figure 1C** and **D**). Similarly, exogenous TGF-β1 treatment of HT22 cells led to the appearance of senescence markers, including an increase in SA-β-gal-positive cells and P16 protein upregulation (**Figure 1E** and **F**). Additionally, increased SMAD2 phosphorylation expression was observed (**Figure 1G**). These findings further confirm that TGF-β1–SMAD2/3 signaling pathway activation promotes neuronal senescence.

### Characteristics of senescent cells after spinal cord injury

**Figure 2:**
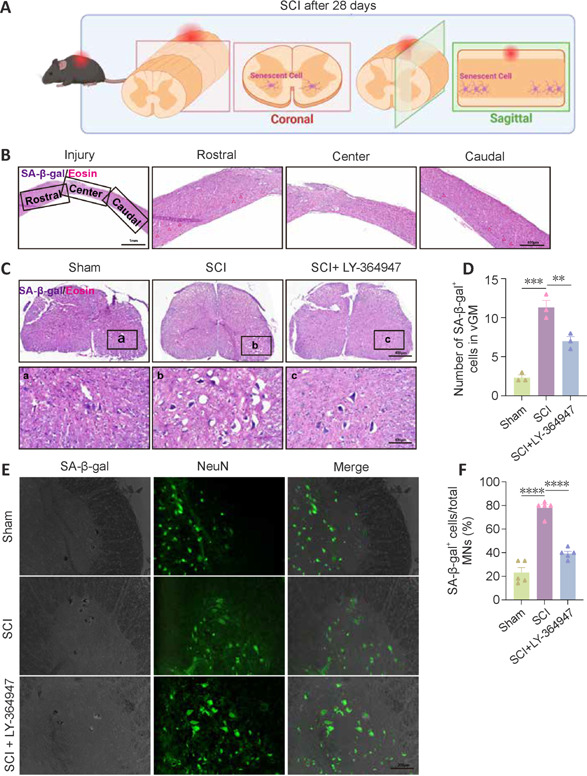
Characteristics of senescent cells at 4 weeks post-SCI. (A) Schematic diagram of sagittal and coronal sections of mouse spinal cord tissue at 4 weeks post-SCI. Created with BioRender.com. (B) Representative images of sagittal spinal cord tissue sections stained with β-galactosidase and H&E at 4 weeks post-SCI. Senescent neurons exhibit blue staining, along with an increased cell volume and a rounded morphology. Scale bar: 400 μm. (C) Representative images of coronal spinal cord tissue sections stained with β-galactosidase and H&E at 4 weeks post-SCI. Scale bars: 400 μm (upper); and 100 μm (lower). (D) Quantitative analysis of the number of senescent cells in the vGM of the spinal cord at 4 weeks post-SCI in mice (*n* = 3). (E) Representative cross-sectional images of spinal cord tissue stained with β-galactosidase (gray) and NeuN (green, Alexa Fluor 488) immunofluorescence at 4 weeks post-SCI. Scale bar: 200 μm. (F) Quantitative analysis of the SA-β-gal positive rate in NeuN^+^ cells in the anterior horn of the spinal cord in different groups at 4 weeks post-SCI (*n* = 5). Data are expressed as mean ± SEM. **P < 0.01, ***P < 0.001, ****P < 0.0001 (one-way analysis of variance followed by Tukey’s *post hoc* test). H&E: Hematoxylin and eosin; LY-364947: a SMAD2 phosphorylation inhibitor; MN: motor neuron; SA-β-gal: senescence-associated β-galactosidase; SCI: spinal cord injury; SMAD2: mothers against decapentaplegic homolog 2; vGM: ventral gray matter.

To determine the senescent cell distribution at 4 weeks post-SCI, sagittal and coronal sections were analyzed using H&E staining and β-galactosidase staining to identify senescent cells (**Figure 2A**). In sagittal sections, we observed senescent cells primarily localized in the ventral gray matter of the spinal cord, with a higher density flanking both sides of the lesion epicenter, while the injury core lacked senescent cells (**Figure 2B**). Coronal sections showed senescent cells predominantly distributed in the anterior horn of the spinal cord, which is characterized by larger cell bodies (**Figure 2C**). Inhibiting SMAD2 phosphorylation resulted in a reduced number of senescent cells in the anterior horn (**Figure 2D**). This suggests that senescent cell accumulation after SCI may be mediated by TGF-β1–SMAD2/3 signaling pathway activation. To further define the senescent cell phenotype, immunofluorescence staining for the neuronal marker, NeuN, was performed following β-galactosidase staining. SA-β-gal and NeuN co-expression in the majority of senescent cells (**Figure 2E**) confirmed that neurons, particularly motor neurons, were the primary senescent cell population. Altogether, these findings suggest that senescent motor neurons in the anterior horn may significantly contribute to motor dysfunction following SCI, and inhibiting SMAD2 phosphorylation results in a lower proportion of SA-β-gal^+^/NeuN^+^ cells (**Figure 2F**).

### Expression of key protein following spinal cord injury

**Figure 3:**
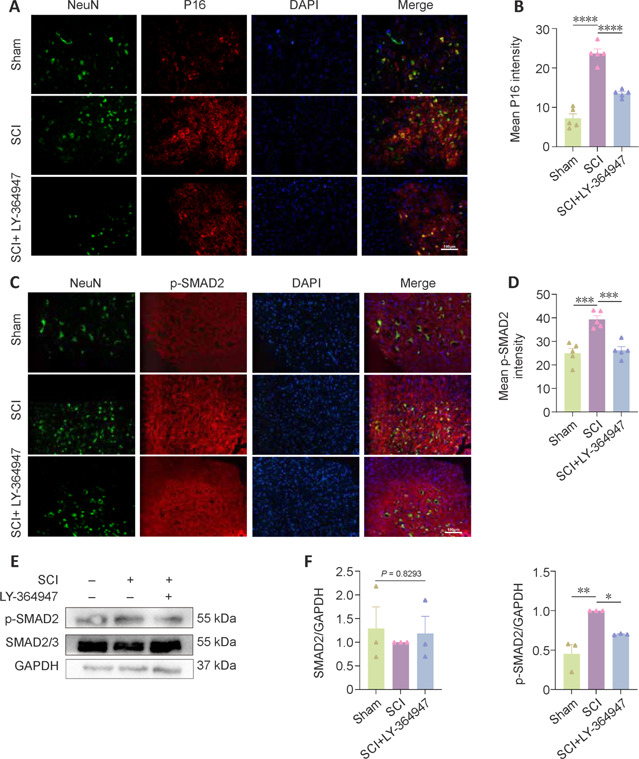
Protein expression profile at 4 weeks post-SCI. (A) Representative images of NeuN (green, Alexa Fluor 488) and P16 (red, Cy3) immunofluorescence in each group at 4 weeks post-SCI (*n* = 5). Scale bar: 100 μm. (B) Relative average intensity of P16 fluorescence in each group at 4 weeks post-SCI. (C) Representative images of NeuN (green, Alexa Fluor 488) and p-SMAD2 (red, Cy3) immunofluorescence in each group at 4 weeks post-SCI (*n* = 5). Scale bar: 100 μm. (D) Relative average intensity of p-SMAD2 fluorescence in each group at 4 weeks post-SCI. (E) Representative images of western blot analysis for SMAD2/3 and p-SMAD2 at 4 weeks post-SCI. (F) Quantitative analysis of SMAD2/3 and p-SMAD2 (*n* = 3). Data are expressed as mean ± SEM. *P < 0.05, **P < 0.01, ***P < 0.001, ****P < 0.0001 (one-way analysis of variance followed by Tukey’s *post hoc* test). DAPI: 4′,6-Diamidino-2-phenylindole; GAPDH: glyceraldehyde-3-phosphate dehydrogenase; LY-364947: a SMAD2 phosphorylation inhibitor; p-SMAD2: phospho-SMAD2; SCI: spinal cord injury; SMAD2/3: mothers against decapentaplegic homolog 2/3.

An increase in senescent neurons was observed following SCI. To determine whether the protein expression of senescent neurons *in vivo* is consistent with *in vitro* neuronal senescence, immunofluorescence staining for P16 and p-SMAD2 was conducted. P16 and NeuN immunofluorescence showed an increase in P16 expression around the injury center at 4 weeks after SCI (**Figure 3A** and **B**). Similarly, SMAD2 phosphorylation and NeuN immunofluorescence showed increased SMAD2 phosphorylation expression around the injury site at 4 weeks post-injury (**Figure 3C** and **D**). Inhibiting SMAD2/3 phosphorylation resulted in a decrease in SMAD2 phosphorylation expression, accompanied by a reduction in P16 expression. This indicates that SMAD2 phosphorylation in neurons induces cellular senescence following SCI. Blocking this pathway may slow the aging process. Western blot analysis also demonstrated that while SMAD2/3 expression remained unchanged, SMAD2 phosphorylation expression increased after SCI, confirming a consistent protein expression of neuronal senescence *in vivo* post-SCI with that observed *in vitro* (**Figure 3E** and **F**).

### SMAD2 phosphorylation inhibitor promotes histological recovery after spinal cord injury

**Figure 4:**
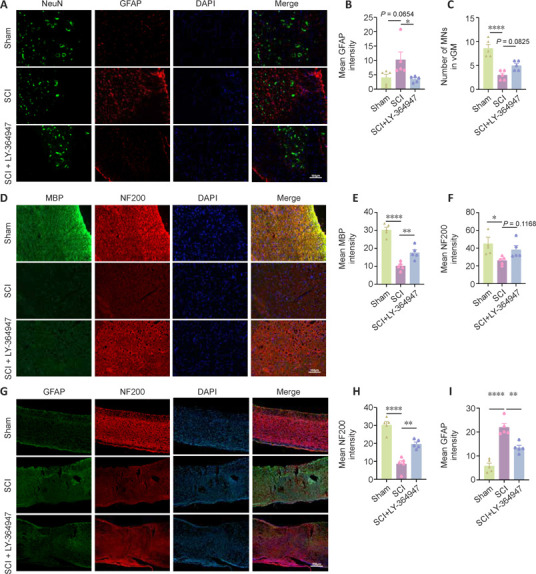
TGF-β1 inhibitor promotes histological recovery at 4 weeks post-SCI. (A) Representative immunofluorescence images of NeuN (green, Alexa Fluor 488) and GFAP (red, Cy3) at the injury margins at 4 weeks post-SCI (*n* = 5). Scale bar: 100 μm. (B) Relative mean GFAP fluorescence intensity at the injury margins at 4 weeks post-SCI. (C) Quantitative analysis of neuronal count in vGM at the injury margins at 4 weeks post-SCI. (D) Representative immunofluorescence images of MBP (green, Alexa Fluor 488) and NF200 (red, Cy3) at the injury margins at 4 weeks post-SCI (*n* = 5). Scale bar: 100 μm. (E) Relative mean MBP fluorescence intensity at the injury margins at 4 weeks post-SCI. (F) Relative mean NF200 fluorescence intensity at the injury margins at 4 weeks post-SCI. (G) Representative immunofluorescence images of GFAP (green, Alexa Fluor 488) and NF200 (red, Cy3) at the injury center at 4 weeks post-SCI (*n* = 5). Scale bar: 200 μm. (H) Relative mean NF200 fluorescence intensity at the injury center at 4 weeks post-SCI. (I) Relative mean GFAP fluorescence intensity at the injury margins 4 weeks post-SCI. Data are expressed as mean ± SEM. *P < 0.05, **P < 0.01, ****P < 0.0001 (one-way analysis of variance followed by Tukey’s *post hoc* test). DAPI: 4′,6-Diamidino-2-phenylindole; GFAP: glial fibrillary acidic protein; LY-364947: a SMAD2 phosphorylation inhibitor; MBP: myelin basic protein; NF200: neurofilament 200; SCI: spinal cord injury; SMAD2: mothers against decapentaplegic homolog 2; TGF-β1: transforming growth factor-β1; vGM: ventral gray matter.

We have demonstrated that inhibiting the TGF-β1–SMAD2/3 pathway can effectively reduce the number of senescent cells and decrease senescence-associated protein expression. However, whether neuronal senescence suppression contributes to tissue repair following SCI remains to be fully elucidated. To address this, we examined tissue-level recovery at 4 weeks post-SCI using immunofluorescence staining. NeuN and GFAP immunofluorescence staining (**Figure 4A**) revealed that inhibiting the TGF-β1–SMAD2/3 pathway at the injury margins significantly reduced astrocyte-mediated glial scar formation (**Figure 4B**) and increased neuronal survival (**Figure 4C**). In addition, MBP and NF200 immunofluorescence staining (**Figure 4D**) showed that blocking this pathway enhanced myelin preservation (**Figure 4E**) and protected neuronal axons (**Figure 4F**). Moreover, in the injury center, GFAP and NF200 immunofluorescence staining (**Figure 4G**) also showed reduced glial scar formation and increased axonal preservation (**Figure 4H** and **I**).

These findings suggest that the presence of a large number of senescent cells post-SCI impedes tissue repair by not only reducing neuronal survival and inhibiting axonal regeneration, but also exerting detrimental effects on astrocytes and oligodendrocytes. This adverse impact may stem from the release of inflammatory factors by senescent neurons. In summary, targeting neuronal senescence through inhibiting the SMAD2 phosphorylation pathway holds promise for enhancing tissue repair and functional recovery following SCI.

### SMAD2 phosphorylation inhibitor promotes recovery of bladder and motor functions after spinal cord injury

**Figure 5:**
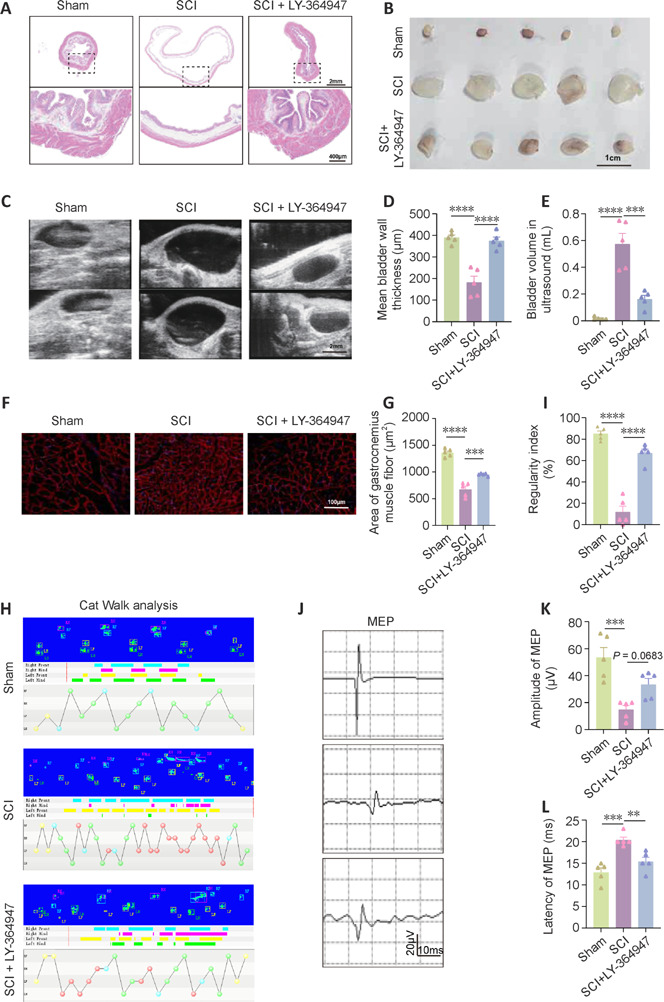
TGF-β1 inhibitor promotes the recovery of bladder and motor functions at 4 weeks post-SCI. (A) Representative H&E-stained images of bladder walls from different groups. Scale bars: 2 mm (upper); 400 μm (lower). (B) Representative digital images show the bladder volume sizes of different groups. Scale bar: 1 cm. (C) Representative ultrasound images showing morphological changes in bladders across different groups. Scale bar: 2 mm. (D) Quantitative analysis of bladder wall thickness across different groups (*n* = 5). (E) Quantitative analysis of residual urine volume in the bladder across different groups (*n* = 5). (F) Representative laminin (red, Cy3) stained images of the gastrocnemius muscle from different groups. Scale bar: 100 μm. (G) Quantitative analysis of gastrocnemius muscle fiber cross-sectional area across different groups (*n* = 5). (H) Representative CatWalk gait analysis images from different groups. (I) Quantitative analysis of regularity index from CatWalk gait analysis across different groups (*n* = 5). (J) Representative MEP images from different groups. (K, L) Quantitative analysis of MEP amplitude and latency across different groups (*n* = 5). Data are expressed as mean ± SEM. **P < 0.01, ***P < 0.001, ****P < 0.0001 (one-way analysis of variance followed by Tukey’s *post hoc* test). H&E: Hematoxylin and eosin; LY-364947: a SMAD2 phosphorylation inhibitor; MEP: motor-evoked potential; SCI: spinal cord injury; SMAD2: mothers against decapentaplegic homolog 2; TGF-β1: transforming growth factor-β1.

To examine functional recovery at 4 weeks after SCI in mice, we examined both bladder function and hind limb motor function. H&E staining found a thinner bladder wall in the SCI group, whereas bladder wall thickness in the LY-364947-treated group was comparable to the sham group (**Figure 5A**). Mice in the SCI group exhibited marked urinary retention, with digital imaging revealing significantly enlarged bladders compared with the sham group. Mice treated with LY-364947 showed a notable reduction in bladder size (**Figure 5B**). Ultrasound measurements indicated a significant increase in residual urine volume in the SCI group, whereas the LY-364947 group displayed a reduction in residual urine, suggesting improved bladder function recovery in the latter (**Figure 5C**). Statistical analysis showed significant recovery of bladder wall thickness and urinary retention in LY-364947-treated mice compared with the SCI group (**Figure 5D** and **E**). SCI causes significant hind limb motor deficits, which are often accompanied by muscle atrophy that severely impacts mobility and function (Kok et al., 2024). Laminin immunofluorescence staining was used to examine the cross-sectional area of gastrocnemius muscle fibers. Mice in the SCI group displayed a marked reduction in muscle fiber cross-sectional area, indicating severe muscle atrophy. While muscle atrophy was still present in the LY-364947 group, it was notably less severe compared with the SCI group (**Figure 5F** and **G**). CatWalk gait analysis revealed significant improvements in hind limb coordination and regularity index in LY-364947-treated mice (**Figure 5H** and **I**). Furthermore, electrophysiological recordings demonstrated reduced latency and increased amplitude in the LY-364947 group compared with the SCI group, indicating enhanced neural function recovery (**Figure 5J–L**).

These findings suggest that treatment with LY-364947 significantly promotes functional and neurological recovery following SCI in mice, as evidenced by improved bladder function, reduced muscle atrophy, and enhanced motor and neural function. This highlights LY-364947 as a promising therapeutic candidate for SCI.

### Transcriptome sequencing of the spinal cord after spinal cord injury and LY-364947 treatment

**Figure 6:**
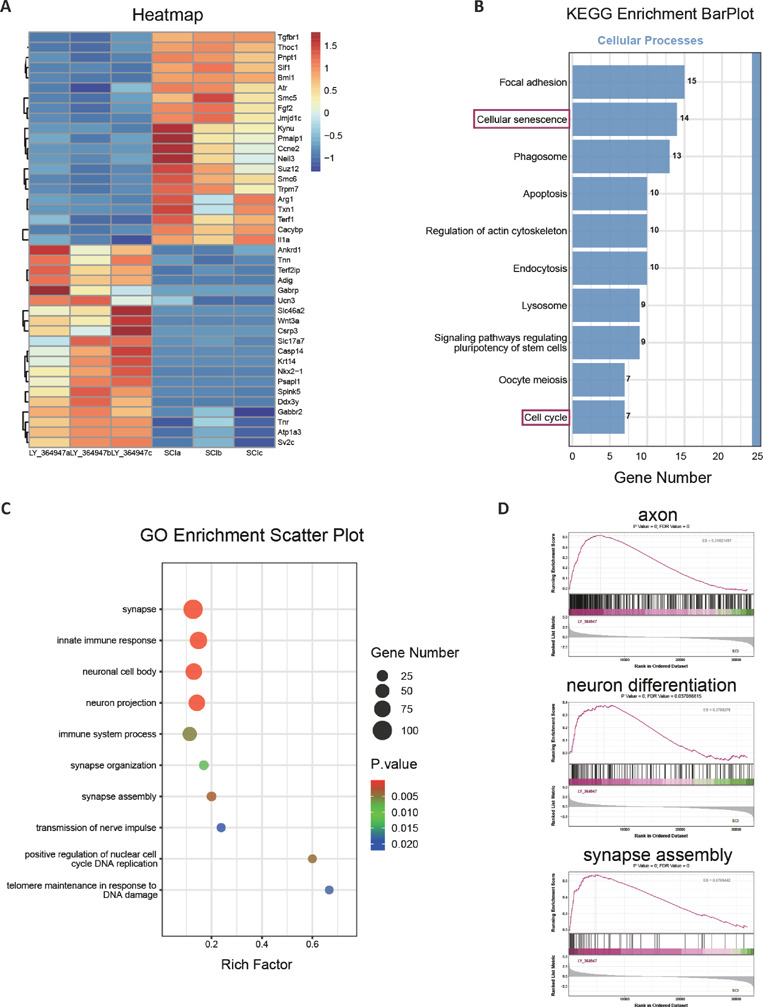
Transcriptome sequencing of the spinal cord at 4 weeks after spinal cord injury and LY-364947 treatment. (A) Heatmap analysis of RNA sequencing throughout the spinal cord injury center in mice (*n* = 3). (B) KEGG pathway analysis shows functional enrichment of RNA sequencing throughout the spinal cord injury center in mice (*n* = 3). The red box is associated with cellular senescence. (C) GO analysis shows gene functional categorization of RNA sequencing throughout the spinal cord injury center in mice (*n* = 3). (D) GSEA indicates gene set enrichment of RNA sequencing throughout the spinal cord injury center in mice (*n* = 3). GO: Gene Ontology; GSEA: Gene Set Enrichment Analysis; KEGG: Kyoto Encyclopedia of Genes and Genomes; LY-364947: a SMAD2 phosphorylation inhibitor; SMAD2: mothers against decapentaplegic homolog 2.

So far, we have shown that inhibiting SMAD2 phosphorylation reduces senescent cells throughout the SCI center and promotes neural repair. However, the specific impact of this inhibition on gene expression remains unclear. To address this, we conducted a comprehensive transcriptome RNA sequencing analysis of spinal cord tissue from SCI margins. Heatmap analysis found that genes associated with cellular senescence, such as *Tgfbr1*, *Atr*, and *Smc5*, were significantly downregulated in the LY-364947-treated group, while genes related to neural repair, such as *Sv2c*, *Atp1a3*, and *Tnr*, were significantly upregulated (**Figure 6A**) (Miyatake et al., 2021; Liu et al., 2023; Preininger et al., 2023; Vasilijic et al., 2023; Zeng et al., 2023). KEGG pathway analysis found significant enrichment of pathways related to cellular processes, particularly cellular senescence and the cell cycle (**Figure 6B**). Following LY-364947 treatment, the expression of genes associated with cellular senescence and cell cycle arrest, such as *Trpm7*, *Sirt1*, and *Tgfbr1*, was notably decreased, with cell cycle arrest being a hallmark of cellular senescence. GO analysis further demonstrated that differentially expressed pathways were predominantly enriched in axon-related processes, innate immunity, neural composition, and DNA damage. The expression changes of related genes, including *Clec5a*, *Ighv1-64*, *Hcn2*, *Nectin1*, *Rock1*, and *Atrx*, confirmed the profound impact of LY-364947 on cellular activity following SCI (**Figure 6C**). Moreover, GSEA analysis revealed significant upregulation of pathways related to neural differentiation, axon assembly, and synaptic functions in LY-364947-treated mice (**Figure 6D**), with the involved genes also highlighted in KEGG and GO analyses.

### Transforming growth factor-beta 1 is primarily released by macrophages after spinal cord injury

**Figure 7:**
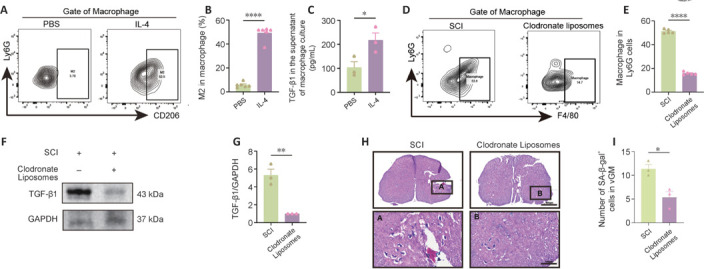
TGF-β1 is primarily released by macrophages at 4 weeks post-SCI. (A) Representative flow cytometry images of macrophages under different intervention conditions. (B) Proportion of M2 polarization in macrophages after *in vitro* PBS and IL-4 interventions. (C) TGF-β1 secretion by macrophages under different intervention conditions. (D) Representative flow cytometry images of macrophages at 4 weeks post-SCI. (E) Quantitative analysis of macrophage proportions in each group at 4 weeks post-SCI. (F) Representative western blot images of TGF-β1 at 4 weeks post-SCI. Scale bars: 400 μm (upper); and 100 μm (lower). (G) Quantitative analysis of TGF-β1 (*n* = 3). (H) Representative sagittal section images of β-galactosidase and H&E staining of mice spinal cord tissue at 4 weeks post-SCI. (I) Quantitative analysis of β-galactosidase-positive cells in coronal sections of mice spinal cord tissue at 4 weeks post-SCI. Data are expressed as mean ± SEM. *P < 0.05, **P < 0.01, ****P < 0.0001 (two-tailed Student’s *t*-test). GAPDH: Glyceraldehyde-3-phosphate dehydrogenase; H&E: hematoxylin and eosin; IL-4: interleukin-4; PBS: phosphate buffered saline; SCI: spinal cord injury; TGF-β1: transforming growth factor-β1.

In studies on natural aging of the spinal cord, it has been shown that mice lack the microglia in primates that promote motor neuron aging. However, in liver injury models, macrophages were found to secrete TGF-β1, accelerating neuronal aging (Bird et al., 2018; Sun et al., 2023). To further investigate whether macrophages in mice can similarly promote motor neuron aging through TGF-β1 secretion after SCI, an *in vitro* experiment was conducted. Macrophages were polarized using IL-4 and flow cytometry used to examine polarization (**Figure 7A**). The results showed that the proportion of polarized macrophages in the IL-4 group increased by 45% compared with the PBS group (**Figure 7B**). Enzyme-linked immunosorbent assay detected a two-fold increase in TGF-β1 secretion in the IL-4 group compared with the PBS group (**Figure 7C**). For *in vivo* experiments, clodronate liposomes were used to deplete macrophages and determine their role in TGF-β1-mediated neuronal aging. Flow cytometry analysis at 28 days post-SCI showed that the number of macrophages in the clodronate liposome-treated group decreased by 75% (**Figure 7D** and **E**). Western blot analysis indicated that TGF-β1 expression in the clodronate liposome group was reduced by 80% (**Figure 7F** and **G**). Additionally, H&E staining and β-galactosidase staining of sagittal spinal cord tissue sections identified a significant reduction in the number of senescent cells after macrophage depletion (**Figure 7H** and **I**). These findings suggest that macrophage activation following SCI may contribute to motor neuron aging through TGF-β1 secretion.

## Discussion

Our study demonstrates that following SCI, senescent neurons accumulate in the anterior horn at both the rostral and caudal regions of the injury site. Using low concentrations of H_2_O_2_ *in vitro*, we successfully established a model of neuronal senescence. Using this model, we found senescence was closely associated with TGF-β1-induced SMAD2 phosphorylation. *In vivo* inhibition of SMAD2 phosphorylation in neurons using LY-364947 led to a significant reduction in the number of senescent neurons. Concurrently, a decrease in senescent cells improved histological features of the spinal cord, evidenced by increased neuronal preservation, reduced scarring, and enhanced axonal and myelin retention. Functionally, mice demonstrated marked improvements in both bladder and motor functions. Furthermore, macrophage depletion after SCI significantly reduced TGF-β1 levels, which was accompanied by a reduced number of senescent neurons. These findings underscore the critical role of macrophage polarization and TGF-β1 secretion in driving motor neuron senescence in the anterior horn following SCI.

The role of senescent cells in central nervous system injury and repair is complex and context-dependent (Preininger et al., 2023). Although both this study and others have confirmed that senescent neuron accumulation in the spinal cord is detrimental, senescent cells exert broader effects beyond directly impacting neural function (Paramos-de-Carvalho et al., 2021). Through the senescence-associated secretory phenotype (SASP), they can influence surrounding cells, leading to increased scarring and reduced myelin retention as senescent cells accumulate. These effects are closely associated with the activity of astrocytes and oligodendrocytes (Fan et al., 2019; Hou et al., 2022). However, studies on zebrafish have shown that senescent cells decrease over time after injury, and the early clearance of senescent neurons during SCI in zebrafish can actually hinder neural repair (Paramos-de-Carvalho et al., 2021). Similarly, in studies of axolotl limb regeneration, neonatal mice heart, and zebrafish heart and fins, the transient presence of senescent cells promotes tissue regeneration (Yun et al., 2015; Sarig et al., 2019; Da Silva-Álvarez et al., 2020). This indicates that under certain conditions, senescent cells can facilitate tissue regeneration, depending on their timely removal. In the context of SCI, current approaches should primarily focus on the clearance of senescent cells (Kirkland and Tchkonia, 2020). However, the long-term goal should be to develop strategies that either more selectively eliminate senescent cells or harness their beneficial effects while mitigating their detrimental impacts. This would allow for a more refined approach to managing cellular senescence of SCI (Yousefzadeh et al., 2021).

The interaction between macrophages and senescent neurons following SCI also warrants further investigation (Zhou et al., 2024). The optimal timing for senescent cell clearance remains unclear, and further research is needed to achieve more precise therapeutic interventions. In axolotl limb regeneration and zebrafish fin regeneration, macrophages are responsible for clearing senescent cells (Yun et al., 2015; Sarig et al., 2019). However, in cases of liver and SCI, macrophage polarization can promote senescence in hepatocytes and neurons, respectively. As one of the most essential immune cells within the central nervous system, the importance of macrophages is undeniable (Prinz et al., 2021). The differing roles of macrophages in these contexts in mammals warrants further investigation. Indeed this could deepen our understanding of the interplay between senescent cells and immune cells in the central nervous system, which could provide insights into the treatment of diseases such as Alzheimer’s disease, multiple sclerosis, and other neurodegenerative disorders.

Although TGF-β1 promotes neural repair in many studies (Krupinski et al., 1996; Buss et al., 2008), its role in promoting cellular senescence is increasingly recognized, as observed in cases of liver injury and the aged spinal cord (Bird et al., 2018; Sun et al., 2023). The varying effects of TGF-β1 in different studies may be attributed to the timing of its action. In the early stages of injury, TGF-β1 can regulate inflammation and reduce neuronal apoptosis. However, as time progresses and the microenvironment around the injury site shifts from “pro-inflammatory” to “anti-inflammatory,” the sustained release of TGF-β1 may induce neuronal senescence. This may be related to the timing and amount of TGF-β1 secretion. Following SCI, in addition to extensive cellular activity, changes in various molecular profiles are equally critical (Hu et al., 2023). A hallmark feature of senescent cells is alteration of their secretory profile, marked by the release of large amounts of SASP (Birch and Gil, 2020). These SASP components play significant roles across diverse physiological and pathological processes. Thus, investigating the SASP secreted by senescent cells after SCI could be key to understanding the complex post-injury microenvironment, representing a promising therapeutic direction for SCI (Ouvrier et al., 2024).

Our findings indicate that SCI-induced senescent cells are predominantly motor neurons in the anterior horn, which aligns with observations in the aged spinal cord (Sun et al., 2023). We observed minimal senescence in other types of neurons or cells in different regions. Understanding why certain cells are spared from senescence under the same microenvironmental conditions could provide insights into preventing motor neuron senescence. This knowledge may help mitigate age-related motor function decline and enhance recovery of motor function in SCI patients (Jo and Perez, 2020).

Our study demonstrates that macrophage-derived TGF-β1 is a significant contributor to neuronal senescence following SCI. Inhibiting SMAD2 phosphorylation or depleting macrophages reduces the number of senescent neurons, promotes neural tissue repair, and accelerates functional recovery. However, the post-injury microenvironment of the spinal cord is highly complex and our interventions are not entirely precise. Determining the optimal timing for SMAD2 phosphorylation inhibition or macrophage depletion, and validating the safety of these interventions will be critical for clinical applications. Therefore, there is still a considerable gap before these approaches can be applied clinically. Nevertheless, targeting the timely removal of senescent cells will be a crucial focus for future SCI treatment strategies. This study has two primary limitations that warrant consideration. First, the *in vitro* experiments used HT22 hippocampal neurons, which may not fully replicate spinal motor neuron responses, thereby limiting translational relevance. Second, murine models fail to recapitulate the complex pathological microenvironment of human chronic SCI, particularly long-term dynamic changes such as immune microenvironment remodeling and age-related cellular interactions. Despite these constraints, our findings confirm that macrophage-derived TGF-β1 drives neuronal senescence post-SCI via SMAD2 phosphorylation, highlighting a potential therapeutic target for mitigating secondary neurodegeneration. Future studies should prioritize human-derived motor neuron models and advanced *in vivo* systems to bridge these translational gaps.

## Additional files:

***Additional Figure 1:*** *Flow cytometry of macrophage identification.*

Additional Figure 1 Flow cytometry of macrophage identification.
Extracted cells showed a CD11B^+^ and F4-80^+^ rate of 97.6%, confirming them as macrophages.

***Additional Figure 2:*** *Timeline of in vivo and in vitro experiments.*

Additional Figure 2 Timeline of *in vivo* and *in vitro* experiments.
(A) Experimental timeline of HT22 cell section. (B) Experimental timeline of LY-364947 intervention post-SCI. (C) Experimental timeline of macrophage section. (D) Experimental timeline of clodronate liposome intervention post-SCI. GFAP: glial fibrillary acidic protein; H_2_O_2_: hydrogen peroxide; HE: hematoxylin and eosin; IL-4: interleukin-4; LY-364947: a SMAD2 phosphorylation inhibitor; MBP: myelin basic protein; NF200: neurofilament 200; PBS: phosphate buffered saline; p-SMAD2: phospho-SMAD2; SCI: spinal cord injury; SMAD2/3: mothers against decapentaplegic homolog 2/3; TGF-β1: transforming growth factor-β1.

## Data availability statement:

*All data relevant to the study are included in the article or uploaded as Additional files.*
